# Neonatal encephalopathy in Aotearoa New Zealand: a review utilising two existing population datasets

**DOI:** 10.3389/fped.2025.1476692

**Published:** 2025-04-15

**Authors:** Malcolm R. Battin, Lynn Sadler, Jutta van den Boom

**Affiliations:** ^1^ Newborn Service, Health New Zealand, Te Whatu Ora, Auckland City Hospital, Auckland, New Zealand; ^2^ Women’s Health, Health New Zealand, Te Whatu Ora, Auckland City Hospital, Auckland, New Zealand; ^3^ Newborn Service, Health New Zealand, Te Whatu Ora, Waikato Hospital, Hamilton, New Zealand

**Keywords:** perinatal asphyxia, encephalopathy, therapeutic hypothermia, cooling, neonatal encephalopathy, hypoxic ischaemic encephalopathy

## Abstract

**Introduction:**

In Aotearoa New Zealand, the Perinatal & Maternal Mortality Review Committee (PMMRC) collects national data on moderate/severe neonatal encephalopathy (NE), including demographic and clinical details, such as treatment with therapeutic hypothermia (TH). The Australian and New Zealand Neonatal Network (ANZNN) collects data on infants who receive TH. However, for ANZNN, receipt of TH is the entry criteria, not severity of NE or gestation. As these datasets have different entry criteria, there is potential to combine and gain greater insight into treatment provided nationally for NE.

**Methods:**

The specific objectives were (1) to compare the NE dataset collected by the PMMRC and the ANZNN cooling dataset from all level 2 and 3 neonatal intensive care units (NICUs) in NZ to understand differences, including the ability to estimate rates of NE over time; (2) to review temporal trends in the provision of TH nationally in NZ over the 9-year period (2010–2018), including documenting the number/year in both datasets and between centre variations; and (3) to assess receipt of TH for NZ infants with moderate to severe NE to ensure it was appropriate and equitable across all groups. The ANZNN dataset is collected in a de-identified manner so analysis was at the aggregate (i.e., total national and/or tertiary NICU) not individual level.

**Results:**

A total of 601 term infants were identified from the PMMRC dataset and 614 term infants from the ANZNN dataset for the study period. The distribution of sex, birth weight, mode of birth, gestation, and plurality were similar between the two datasets. However, there was a difference in the numbers by year of birth. ANZNN demonstrated a trend towards more infants over time consistent with greater use of TH. However, PMMRC demonstrated a stable proportion of infants receiving TH.

**Conclusion:**

The combined data enabled an estimate to be made of the total NE burden nationally. Moderate and severe NE was documented over the epoch using the consistent PMMRC criteria but the additional ANZNN data illustrated infants cooled outside of moderate to severe NE. The two datasets were definitely not interchangeable for the purpose of NE case ascertainment. There were no major differences demonstrated in the receipt of TH when analysed by ethnicity.

## 1 Introduction

Neonatal encephalopathy (NE) refers to a “clinical syndrome of disturbed neurologic function in the first week after birth in an infant born at or beyond 35 weeks of gestation, manifest by a subnormal level of consciousness or seizures, often accompanied by difficulty with initiating and maintaining respiration plus depression of tone and reflexes” (1, 2). It is an “umbrella term,” which does not imply aetiology. Initially NE typically referred to infants born at term (3); however, in recent years, its use has extended down to include late preterm infants, particularly from 35 weeks of gestation (2). This reflects the routine practice of providing therapeutic hypothermia (TH) for affected infants across this gestational age range (4). The term hypoxic ischaemic encephalopathy (HIE) is appropriately used when there is evidence of hypoxic-ischaemic (HI) insult as a cause of encephalopathy (2, 5).

Worldwide, intrapartum-related events remain major causes of childhood death or significant long-term disability (6–8). Therefore, it is important for maternity and neonatal health systems to develop and implement strategic interventions to reduce associated mortality and morbidity. Such programmes may act through prevention (9, 10) and/or prompt neonatal identification (9, 11) then appropriate mitigation with TH (4). A fundamental first step in development of these strategies is establishing the true size of the problem and then an ability to measure impact from any clinical intervention by ongoing analysis.

In Aotearoa New Zealand (NZ), national data on moderate and severe NE in term infants have been collected by the Perinatal & Maternal Mortality Review Committee (PMMRC) since 2010 (12). In 2016, this data collection was extended to include all infants from 35 weeks of gestation (12). The dataset contains key clinical and demographic details, including receipt of TH, and is reported annually (12) with recommendations for clinical improvement. In addition to this quality reporting function, the data facilitate case review and opportunities for research (13, 14). Key knowledge gained includes identification of intrapartum factors and potential preventability for NE arising in association with (15) and without (16) acute peripartum events. However, the PMMRC dataset uses moderate and severe NE as entry criteria and so does not include infants with mild NE. As umbilical cord gas testing was not universally available, it was collected if available but not considered to be essential entry criteria for the dataset. The original trials of TH were performed in infants with moderate to severe HIE (17–20) and built a strong evidence base for the benefit of TH in this group (4). Notwithstanding this evidence base, there have been several reports of therapeutic drift with TH being provided for mild NE (21–23) and occasionally rewarming before 72 h (24). As mild NE is not captured in the PMMRC dataset, it is important to review other available data to more fully understand the current range of practice and scope for clinical practice improvement in NZ. An obvious complementary dataset is that compiled by the Australian and New Zealand Neonatal Network (ANZNN) (25), which collects data on infants requiring neonatal intensive care to provide quality assurance including all infants who have received TH. Notably, the ANZNN dataset differs from the PMMRC in inclusion criteria, with receipt of TH as the primary entry criteria, not severity of NE or gestational age. As these two datasets have different entry criteria and collect different items, the combination may give greater insight into the clinical burden and treatment provided for NE in a NZ context. Specifically, infants with mild NE who receive TH will be reported to ANZNN but are not captured in the PMMRC dataset. Thus, an increase in use of TH for mild encephalopathy nationally may be reflected in the number and clinical characteristics of infants reported to ANZNN over time but may not be evident in PMMRC data. Indeed, internationally, an increased use of TH for infants with mild HIE has been associated with less severe clinical characteristics in data from the UK (26). In addition, high rates of TH are reported for mild NE in cohorts from the USA (27). Conversely infants who are deemed too unwell to receive TH, die before admission to the neonatal unit, arrive too late or have other reasons not to receive TH, such as infection, will be entered into the PMMRC dataset but will not appear in the ANZNN dataset. Thus, changes in case rate and severity as a result of preventive strategies may manifest in differing ways across the two datasets.

Accordingly, we planned an analysis to document the similarities, overlap, and differences in characteristics identified from the two datasets, with PMMRC inclusion based on moderate-severe NE and ANZNN inclusion on the receipt of TH. The knowledge gained will ensure an understanding of the characteristics of these two datasets, which can inform both future education and ongoing clinical practice improvement activities regarding NE in NZ. Furthermore, this analysis can assess potential variation in the use of TH resources across tertiary regions and by ethnicity. Finally, these results can inform future planning of neonatal services by extrapolating data from centres with a lower threshold for TH to estimate the impact of guideline change or ongoing clinical drift on national TH resource use.

The specific objectives were to:

(1) Compare the NE dataset collected by the PMMRC and the ANZNN cooling dataset collected from all level 2 and 3 neonatal intensive care units (NICUs) in NZ to understand differences, including the ability to estimate rates of NE over time.
(2) Review temporal trends in the provision of TH nationally in NZ over the 9-year period (2010–2018), including documenting the number/year in both datasets and between centre variations.
(3) Assess receipt of TH for NZ infants with moderate to severe NE to ensure it was appropriate and equitable across all groups.

## 2 Methods

Data from the two prospectively collected datasets were compared to document trends in rates of NE and/or receipt of TH. Where possible, a comparison of the clinical and demographic data from the two datasets was performed. For consistency, the analysis of trends in both datasets has been restricted to infants born at ≥37 weeks of gestation. As the ANZNN dataset is collected in a de-identified manner, the analysis was at the aggregate not individual level, i.e., either as total national numbers or grouped by tertiary NICU, and could not compare data collected on individual cases. The PMMRC data were also provided in a de-identified format including data required to designate the tertiary NICU (i.e., place of birth and transfer information).

### 2.1 Study dataset details

A PMMRC data inclusion criterion is a diagnosis of moderate or severe (Sarnat stage 2 and 3) NE and includes infants who died (at any point including before NICU admission), for the period 2010–2018 inclusive. The cases were notified by a clinician who completed the initial mandatory form for the national data collection, with further information submitted by a PMMRC local coordinator and the mother's lead maternity carer (LMC). The LMC under the NZ Maternity system is a midwife, obstetrician, or general practitioner responsible for care throughout pregnancy, labour, and birth.

ANZNN data inclusion was based on receipt of treatment with TH and admission to any level 3 or 2 NICUs in NZ, for the period 2010–2018 inclusive. The ANZNN data included those infants who died during TH or had hypothermia ceased before 72 h of treatment for clinical reasons, but did not include infants who did not receive TH. As it required admission to a neonatal nursery, it did not include stillborn infants and those who died in the birthing suite before NICU admission. Initially, ANZNN only collected binary yes/no for TH; however, from 2012, onward timing and details of cessation of cooling were added.

The PMMRC dataset contained details from a neonatal form documenting demographics, umbilical cord gas details, resuscitation, neonatal clinical course plus cerebral imaging, and short-term outcome. The maternity information included maternal medical history, antenatal and pregnancy details, including birth mode and time plus transfers and placental findings. Details of the definitions and methods used in this dataset are published (28). To analyse the PMMRC data in a de-identified format, at an aggregate regional level, the appropriate tertiary NICU was allocated from the place of birth unless birth was at the single level 2 plus unit that was known to previously offer TH using gel packs and there was no record of transfer to a tertiary unit (i.e., both items). This process of nominal designation to the appropriate regional tertiary unit was also followed for infants born outside of hospital or those who sadly had an early neonatal death before tertiary NICU admission. The justification for this is that typically a transfer team were attending or decisions were made after discussion with the tertiary team. Similarly, the ANZNN dataset included items on maternal demographics, perinatal complications, labour, and birth. In addition, there were items on neonatal status including Apgar scores, resuscitation, neonatal unit admission, treatment with TH, discharge details, morbidity, and mortality (25).

The data on ethnicity are collected in different ways in the two datasets. Specifically, the PMMRC collects maternal and infant prioritised ethnicity, sourced primarily from birth registration when ethnicity is identified by the parent(s). This process of collecting and reporting ethnicity in New Zealand is in accordance with the Health Information Standards Organisation document (29). Use of this standard procedure is intended to improve the accuracy and consistency of ethnicity data in the New Zealand health sector. However, the ANZNN dataset spans a few countries including Australia, Hong Kong, and Singapore in addition to New Zealand. ANZNN defines ethnicity as: *Ethnic origin of the mother of the baby, as identified by the mother*, sourced from the hospital clinical management system and coded as follows: Aboriginal, Asian, Caucasian, Other, Pacific, and Māori (25). Data are extracted and submitted to the ANZNN repository by the neonatal unit representative.

### 2.2 Statistical analysis

An analysis was undertaken using STATAv17.0 and Epi Info™. Clinical data are presented as median (IQR) or number (%). Where categorical variables were compared, the chi-square test was used. Trends in rates over time were tested using the Mantel–Haenszel chi-square test for linear trend in Epi Info™. The characteristics of individuals in the ANZNN and PMMRC datasets, and a subgroup in the PMMRC dataset who received TH, are described but not compared statistically as the data are not independent, with many individuals appearing in both sets. Finally, based on the initial findings on rate of TH between centres, a secondary analysis of the ANZNN data reported from a unit with lower threshold for TH was compared to the other five units to examine the severity of clinical illness. Statistical significance where tested was set at *p* < 0.05.

### 2.3 Ethics approval

The study was judged outside the scope of HDEC review and so did not require ethical approval. An application was submitted to both PMMRC and ANZNN bodies, and approval given, for use of the data. Approval for research using the dataset was granted by ANZNN Research Review Group and ANZNN Advisory Council. Institutional approval was granted by the Auckland Hospital Research Committee (reference no. ADHB 8702).

## 3 Results

**Figure 1:**
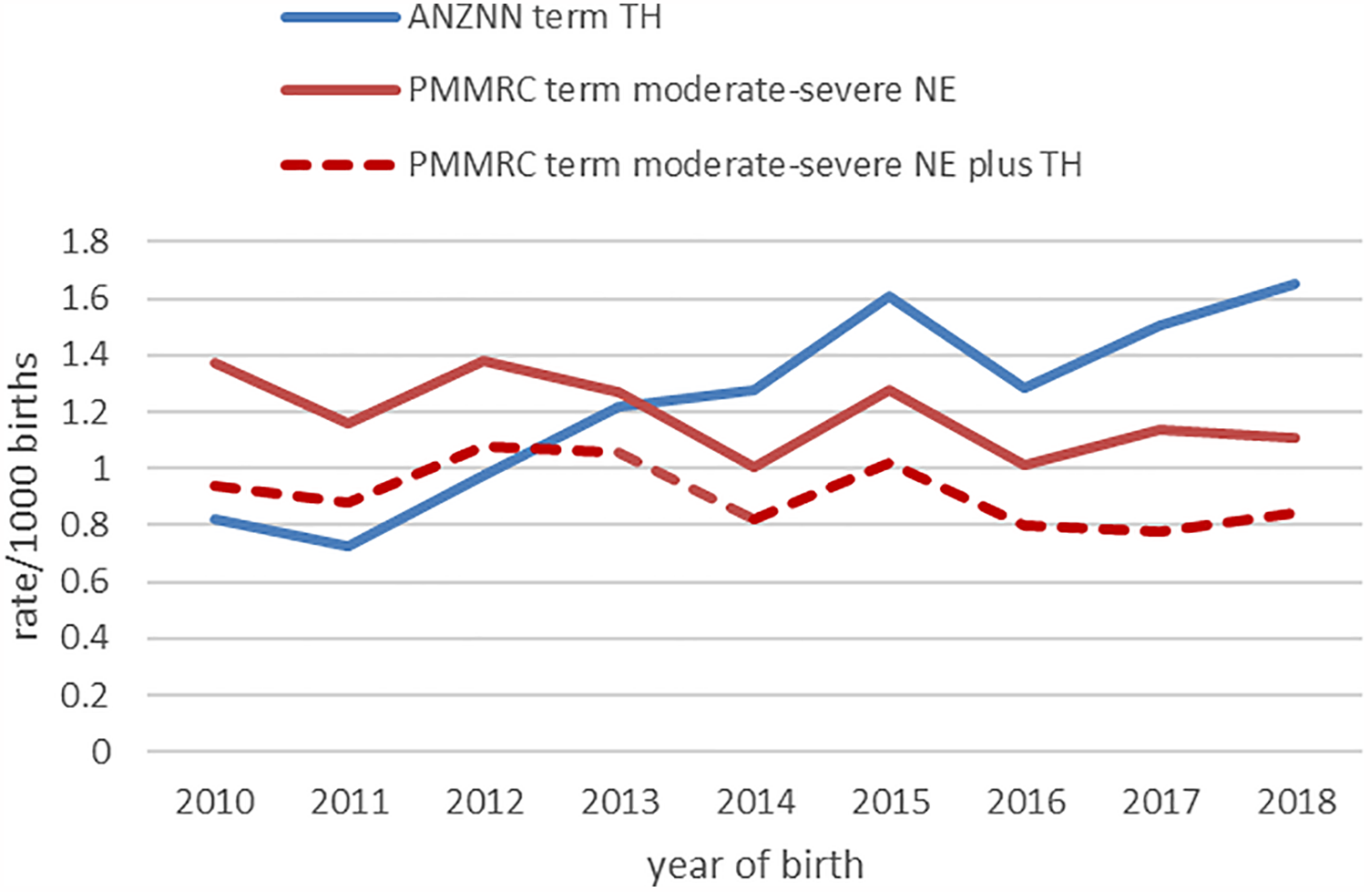
Annual rate of inclusion into the PMMRC (red) and ANZNN (blue) datasets. ANZNN demonstrated a statistically significant increase (*p* < 0.01). ANZNN, Australian and New Zealand Neonatal Network; NE, neonatal encephalopathy; PMMRC, Perinatal & Maternal Mortality Review Committee; TH, therapeutic hypothermia.

For the study period 2010–2018 inclusive, there were 601 term-born infants identified in the PMMRC dataset and 614 term-born infants in the ANZNN dataset. Although these numbers are similar, the cohort differed considerably once the PMMRC data were restricted to just term-born infants who received TH, which numbered just 460 infants. The PMMRC data showed a non-significant decrease over time in cases of infants with moderate to severe NE (*p* = 0.097). However, ANZNN data demonstrated a statistically significant (*p* < 0.01) increase in annual rates in infants receiving TH across the epoch (Figure 1).

The number of infants with NE per year plus details of gestation, birth weight distribution, sex, and plurality for the 9 years of data are documented in Table 1. Based on these data, it is evident that the distribution was similar across the ANZNN data, PMMRC, and PMMRC TH groups. However, comparison of some characteristics, such as maternal age, parity, and body mass index, was not possible as these data items are not available from the ANZNN dataset.

**Table 1:** Details of the year, gestation, birth weight, sex, and plurality of the cohorts.

Characteristic	ANZNN ≥37 weeks; any stage; TH		PMMRC ≥37 weeks; Sarnat 2/3		PMMRC; ≥37 weeks; Sarnat 2/3; TH	
	*n*=	614	*n*=	601	*n*=	460
	*n*	%	*n*	%	*n*	%
Year of birth						
2010	49	8.0	82	13.6	56	12.2
2011	42	6.8	67	11.2	51	11.1
2012	56	9.1	79	13.1	62	13.5
2013	67	10.9	70	11.7	58	12.6
2014	70	11.4	55	9.2	45	9.8
2015	88	14.3	70	11.7	56	12.2
2016	71	11.6	56	9.3	44	9.6
2017	83	13.5	63	10.5	43	9.4
2018	88	14.3	59	9.8	45	9.8
Gestation at birth (weeks)						
37	58	9.5	67	11.2	49	10.7
38	92	15.0	95	15.8	72	15.7
39	135	22.0	137	22.8	103	22.4
40	173	28.2	151	25.1	123	26.7
41	142	23.1	135	22.5	102	22.2
≥42	14	2.3	16	2.7	11	2.4
Birthweight (g)						
<2,500	24	3.9	23	3.8	17	3.7
2,500–3,499	320	52.1	313	52.1	230	50.0
3,500–4,499	249	40.6	243	40.4	196	42.6
≥4,500	21	3.4	22	3.7	17	3.7
Sex						
Male	334	54.4	323	53.7	247	53.7
Female	278	45.3	278	46.2	213	46.3
Unknown	2	0.3		0.0		0.0
Plurality						
Singleton	604	98.4	589	98.0	452	98.3
Twins	10	1.6	12	2.0	8	1.7

ANZNN, Australian and New Zealand Neonatal Network; PMMRC, perinatal and maternal mortality review committee; NE, neonatal encephalopathy; g, grams; TH, therapeutic hypothermia.

Details of available ethnicity information and location of care across the six level 3 neonatal units are presented for the three cohorts in Table 2. On reviewing geographic location, using the ANZNN data on tertiary units, it is evident that one unit has contributed 42.7% of infants receiving TH nationally but only 27.5% of the PMMRC case numbers. The 42.7% finding is higher than expected from the birthing population draining to that unit for level 3 care (circa 25% of national births) and this unit has a recognised lower threshold for TH, consistent with the previously described international practice drift in providing TH to infants with mild NE (26, 27).

**Table 2:** Ethnicity and unit providing care as reported in each dataset.

Characteristic	ANZNN ≥37 weeks; any stage; cooled		PMMRC ≥37 weeks; Sarnat 2/3		PMMRC + TH; ≥37 weeks; Sarnat 2/3; cooled (active)	
	*n*=	614	*n*=	601	*n*=	460
	*n*	%	*n*	%	*n*	%
Ethnicity (mother)						
Māori	128	20.9	157	26.1	118	25.7
Pacific	77	12.5	79	13.1	60	13.0
Asian	80	13.0	78	13.0	66	14.4
Indian	NA		34	5.7	26	5.7
Other Asian	NA		44	7.3	40	8.7
European^a^/Caucasian^b^	313	51.0	277	46.1	210	45.7
NZ European	NA		242	40.3	183	39.8
Other European	NA		35	5.8	27	5.9
Other	12	2.0	10	1.7	6	1.3
MELAA			10	1.7	6	1.3
Unknown	4	0.6		0.0		0.0
Unit						
1	79	12.9	120	20.0	85	18.5
2	72	11.7	78	113.0	67	14.6
3	100	16.3	141	23.5	102	22.2
4	262	42.7	165	27.5	128	27.8
5	55	9.0	64	10.7	52	11.3
6	21	3.4	27	4.5	21	4.6
Level 2 units	25	4.1	4	0.7	4	0.7
Missing data			2	0.3	1	0.2

^a^ The term used in the PMMRC dataset.

^b^ The term used in ANZNN.

Finally, it is noted that the ANZNN data include 25 infants who have had an episode of care assigned to a level 2 (secondary) rather than a level 3 (tertiary) care unit. On closer scrutiny, 10 of these infants were early in the data collection, before 2012, when further details were not collected. It is likely these infants were initially cooled but then clinically improved and were considered to not require ongoing TH. They were then warmed up and not transferred to a level 3 unit. Indeed, a further seven infants, born after 2012, were coded as initially cooled but then warmed up as further TH was not considered to be warranted. Four infants died or were given palliative care at a level 2 unit. The remaining four infants received TH using low technology approaches at a secondary neonatal unit between 2014 and 2017 inclusive. Similarly, four infants born after 2012 were allocated to the level 2 unit in the PMMRC dataset.

Available information on mode of birth, resuscitation requirement, and Apgar scores are documented in Table 3. The mode of birth was similar across the groups, particularly when comparison is made between infants in the ANZNN dataset and the PMMRC dataset, restricted to just those who received TH, with 41.4% versus 43.6%, respectively, born by caesarean section. As the ANZNN dataset did not include parity or acute (sentinel) intrapartum complications, it was not possible to compare the frequency of these across the two datasets. However, the PMMRC data for acute events matched prior published experience (12). It is noted that PMMRC TH had greater frequency of depressed 1-min Apgar but not 5-min Apgar score when compared with the ANZNN cohort. Data comparison for the 10-min Apgar score was not possible as this was not included in the ANZNN dataset. However, for the PMMRC dataset the 10-min Apgar score was below 7 in 318/610 (52%) overall and 285/460 (62%) who received TH. Similarly, resuscitation at birth and components of this were more common in PMMRC TH subset than in the whole PMMRC cohort, which suggests they were more severely affected. Some neonatal clinical characteristics including stage of encephalopathy, resuscitation at birth, inotrope requirement, ventilation, and MRI result were not available for the ANZNN cohort, so it was not possible to compare these across the datasets to estimate the severity of condition. However, nitric oxide use was reported to be 120/460 (26%) and 132/614 (21.5%) in the PMMRC TH and ANZNN cohorts, respectively. Furthermore, as expected due to differences in data collection, there were differences between the two datasets in mortality, which was recorded as 18% in the PMMRC TH and 12% in the ANZNN, respectively.

**Table 3:** Mode of birth, resuscitation, NE severity, and mortality.

Characteristic	ANZNN ≥37 weeks; any stage; cooled		PMMRC ≥37 weeks; Sarnat 2/3		PMMRC; ≥37 weeks; Sarnat 2/3; cooled (active)	
	*n*=	614	*n*=	601	*n*=	460
	*n*	%	*n*	%	*n*	%
Acute complications in labour	NA		154	25.6	136	29.6
Mode of birth						
Normal vaginal birth	239	38.9	242	40.3	176	38.3
Vaginal breech			12	2.0	10	2.2
Operative vaginal	113	18.4	94	15.6	75	16.3
Caesarean section	254	41.4	253	42.1	199	43.3
Elective			11	1.9	4	0.9
Emergency			242	40.3	195	42.4
Apgar 1 min <4	471	76.7	444	73.9	377	82.0
Apgar 5 min <7	569	92.7	460	76.5	386	83.9
Resuscitation at birth			555	92.4	446	97.0
Cardiac massage	NA		238	39.6	197	42.8
IPPV with mask OR ETT	NA		546	90.8	440	95.7
Induced cooling	614	100.0	460	76.6	460	100.0
Severity of NE/markers						
Sarnat stage						
Moderate	NA		414	68.9	331	72.0
Severe	NA		187	31.1	129	28.0
Anticonvulsant requirement (y/n)	NA		427	71.1	327	71.1
Requirement for NO	132	21.5	141	23.6	120	26.1
Died before admission	NA		17	2.8	0	
Death after admission to NICU	74	12.1	120	20.0	84	18.3

ETT, endotracheal tube; IPPV, intermittent positive pressure ventilation; NA, not available; NICU, neonatal intensive care unit; NO, nitric oxide.

A secondary analysis of the ANZNN data reported from unit 4, which has a lower threshold for TH, showed that the infants had less severe clinical illness. Specifically, comparing unit 4 PMMRC TH data with the other centres, death was less frequent (13.3% vs. 20.5%), anticonvulsant treatment was less frequent (64.0% vs. 74.6%), and cardiac massage as part of resuscitation was less frequent (32.8% vs. 47.1%). Similarly, Apgar scores <4 at 1 min were less frequent (78.0% vs. 84.1%).

Finally, as TH is the only postnatal intervention with proven benefit, it is important to examine receipt by ethnicity. The receipt of TH in infants over 37 weeks of gestation was assessed by prioritised infant ethnicity with no significant difference in proportion detected (*p* = 0.3), although numbers were small in some groups (Table 4).

**Table 4:** Receipt of TH by prioritised infant ethnicity using PMMRC data.

Prioritised ethnicity	Total number	Received TH, *n* (%)
Māori	187	139 (74)
Pacific	84	63 (75)
Asian		
Indian	36	28 (77)
Other Asian	40	36 (90)
European		
NZ European	187	139 (74)
Other European	24	20 (83)
Other		
MELAA	10	6 (83)

No difference in receipt of TH on testing with chi square (*p* = 0.3). PMMRC, Perinatal & Maternal Mortality Review Committee; TH, therapeutic hypothermia.

## 4 Discussion

The overarching purpose of this study was to gain an understanding of the relationship between the two available datasets and consider how these could best inform ongoing national quality improvement efforts. Indeed, the combined data enabled a greater insight into the total NE burden nationally, which would not be possible if using just one dataset. Specifically, moderate and severe NE were documented over the period of study using the consistent PMMRC criteria; however, inclusion of the analysis of the ANZNN data added information on infants cooled outside of the moderate to severe NE criteria. Accordingly, there was an improved understanding of both NE burden across the whole range of mild through to severe NE plus data on the corresponding care and services provided. However, the two datasets were shown to definitely not be interchangeable for the purpose of NE case ascertainment. The mismatch of numbers and absence of interchangeability is illustrated by the incongruities between cohorts, which revealed a sizable difference in numbers plus differing trends over time.

It is helpful to consider explanations for the differences between these two datasets. Specifically, the increase in number of infants receiving TH over time in the ANZNN dataset occurred without a concomitant increase in the proportion of NE infants receiving TH or an increase in number of infants diagnosed with NE in the PMMRC data (12). This points towards more infants with mild NE receiving TH, similar to international experience (26, 27, 30), and therefore contributes to the understanding of services provided. Apropos the specific objective of estimating rates of NE overtime, in a NZ national context, using “receipt of TH” as a proxy measure for NE rate would be erroneous and should not be used to assess the impact of national quality and safety programmes aimed at decreasing NE. Further population-specific analyses, using appropriate datasets, would be required before generalising this finding to other countries. However, a similar finding has recently been reported from a population-based cohort from the USA with no significant change in HIE, as defined by a combination of abnormal cord gas and encephalopathy, but increased use of TH observed over an 8-year period (31). Crucially, judging impact from national strategic interventions in any setting would require good case ascertainment and consistency in identifying cases to be included. In addition, clear documentation of any shift in practice must be factored into interpretation, which maybe more difficult if practices vary by unit.

The current analysis also highlighted practice variability between units with regard to intervention with TH for mild encephalopathy. Historically, some professional groups, such as UK BAPM, suggest that TH should not be provided in mild NE outside of a trial (32). Similarly, previous reviews (33) have reported there to be insufficient evidence to recommend TH in mild NE. However, the situation is evolving so our study findings may be used to inform service requirements should there be future practice change. Table 1 shows that the bulk of infants who had mild NE and received TH are principally from one unit, which also contributed the largest proportion of cases with moderate and severe NE to the PMMRC dataset. If national guidelines shifted and TH became the standard of care for mild NE, data stemming from the current analysis could assist in estimating extra resources required. Essentially, in this unit with a lower threshold for cooling the total number of infants who received TH was approximately twice the number with moderate/severe NE and receiving TH.

A further point of consideration is that 141 infants with moderate/severe NE were documented to not receive TH, which equates to 23% of the 601 notified to the PMMRC, and are infants with moderate/severe NE who would not be visible in the absence of this case-based dataset. The PMMRC has previously reviewed infants who were not cooled (34), with reasons cited including early neonatal death, withdrawal of care, disseminated infection, and late presentation or late recognition. These features are consistent with UK data (30) reporting 37.9% of infants diagnosed with moderate or severe HIE did not receive TH, with a diagnosis outside of the TH window and being too sick to cool reported as potential contributing factors. The cohort was quite sick with high rates of NO use for pulmonary hypertension, with use fairly similar across the ANZNN and PMMRC datasets.

Accurate data on demographic factors are important to optimally support research and quality improvement. Specifically, with regard to NE, a relationship with socioeconomic status is recognised in the PMMRC reports (12). Internationally, the offspring of immigrant women have been shown to be at increased risk (35). Furthermore, ethnicity has been reported to be linked with differences in outcome of preterm birth (36) plus other maternal and perinatal outcomes (37) in Aotearoa New Zealand. Although the data collection for ANZNN and PMMRC are not currently aligned due to differences in both inclusion criteria and collection of ethnicity data, an expansion of data collection by ANZNN is planned after a working group review. In the future, this may facilitate a more detailed analysis, particularly if the entry criteria are widened to include moderate and severe NE admitted to NICUs, not just TH receipt. In addition, clinical factors, such as primigravid status (38, 39), or other maternal risk factors are important for NE (40, 41). Therefore, such factors should be considered to appraise any potential contribution when designing interventions to reduce NE rates.

The third aim of the study was to assess receipt of TH in NZ infants with NE to ensure it was appropriate and equitable across all ethnicity groups. The analysis used a clinical diagnosis of moderate/severe NE as a prerequisite to receipt of TH and utilised local ethnicity data, as available in the PMMRC dataset. Reassuringly, there were no major differences demonstrated in the receipt of TH when analysed by ethnicity, although there was a geographical difference, with the higher use of TH for mild NE at one unit. The ongoing review of infants who had moderate/severe NE but did not receive TH is an important ongoing assessment of the system (42).

The current study has several strengths. First, it used national population-based data with probable complete ascertainment, albeit using different inclusion criteria for the two sets. Both datasets are collected for the purpose of driving quality improvement leading to improved neonatal outcomes. Furthermore, it is recognised that the quality of the data is good, with the opportunity to validate the data by local unit on submission to ANZNN and by a practitioner on submission to PMMRC. Finally, both organisations clean the data in preparation for analysis. However, there are study limitations as some data were unavailable in each set. It is also noted that the ANZNN dataset is collected in a de-identified format, which means a comparison cannot be made at the individual level. Given the recognised influence of demographics on healthcare utilisation, with ethnicity an important consideration, future development of the datasets may enable a formal comparison of ethnicity data; however, this was not possible in the current analysis. Finally, the PMMRC data collection is based on a definition of NE, as is common practice (2), rather than requiring evidence of hypoxic ischaemic insult. Although a history of acute sentinel events is recorded and umbilical cord gases are documented where available, it is possible that a small number of included infants had a non-hypoxic aetiology for the encephalopathy.

In conclusion, this study has improved the understanding of NE and provision of service including TH in NZ. The findings will be helpful in informing ongoing data collection specifically aimed at assessing impact of strategies or documenting any current variation in resource use (i.e., cooling). Furthermore, the analysis could inform guideline development and future planning of neonatal services by estimating capacity required with altered (i.e., milder) clinical criteria for cooling. Future work will be also enabled by the increase in data being collected by ANZNN from 2025. Finally, despite recent stress on NICU occupancy in NZ, it is reassuring that there is equitable use of TH in this vulnerable group of infants by ethnicity and at least in the majority of units.

## Data Availability

The datasets presented in this article are not readily available because the datasets are only available through application to the two bodies responsible for data collection and governance. Requests to access the datasets should be directed to ANZNN https://anznn.net and National Mortality Review Committee https://www.hqsc.govt.nz/our-work/national-review-of-avoidable-deaths/national-mortality-review-committee/.
